# Immunological Evidence for the Role of Mycobacteria in Sarcoidosis: A Meta-Analysis

**DOI:** 10.1371/journal.pone.0154716

**Published:** 2016-08-01

**Authors:** Chuling Fang, Hui Huang, Zuojun Xu

**Affiliations:** Department of Respiratory Medicine, Peking Union Medical College Hospital, Chinese Academy of Medical Sciences & Peking Union Medical College, Beijing, China; Universitatsklinikum Freiburg, GERMANY

## Abstract

**Background:**

Sarcoidosis is a granulomatous disease, the etiology of which is currently unknown. The role of mycobacteria in the etiology of sarcoidosis has been extensively investigated. In this meta-analysis, we assessed the immunological evidence of the possible role of mycobacteria in the pathogenesis and development of sarcoidosis.

**Methods:**

We performed a systematic search of relevant articles from PubMed, Embase and Cochrane Library databases published between January 1990 and October 2015. Data extracted from the articles were analyzed with Review Manager 5.3 (Cochrane Collaboration, Oxford, UK).

**Results:**

In this meta-analysis, 13 case-control studies (733 participants) were considered eligible according to our criteria. Methodological quality was assessed using the Newcastle-Ottawa Scale (NOS). The positivity incidence of the immune response (either the cell-mediated response or humoral response) in sarcoidosis patients was significantly higher than that in controls, as determined using fixed-effects model. The odds ratio (OR) of the positivity incidence of T-cell response in the patients with sarcoidosis versus the controls with PPD- or unknown PPD status was 5.54 (95% CI 3.56–8.61); the ORs were 16.70 (95% CI 8.19–34.08) and 1.48 (95% CI 0.74–2.96) for the two subgroups with PPD- controls and unknown PPD status respectively. However, the OR of the positivity incidence in patients with sarcoidosis versus PPD+ controls (latent tuberculosis infection; LTBI) was 0.26 (95% 0.10–0.66). Regarding the humoral response, pooled analysis of the positivity incidence revealed an OR (95%CI) of 20.43 (5.53–75.53) for the patients with sarcoidosis versus controls; the ORs were 11.93 (95% CI 2.15–66.27) and 41.97 (95% CI 5.24–336.15) in two subgroups of controls with PPD- and unknown PPD statuses respectively. Data on heterogeneity and evidence of publication bias were examined.

**Conclusions:**

This meta-analysis confirmed the existence of an association between mycobacteria (especially *M*.*tuberculosis*) and sarcoidosis. The current available evidence indicates that some insoluble mycobacterial antigens that preferentially within the body are involved in the pathogenesis of sarcoidosis rather than the whole mycobacteria and that they elicit a type IV immune response.

## Introduction

Mycobacterium tuberculosis (*M*. *tuberculosis*) is the second most common infectious cause of death in adults worldwide (HIV is the most common). The human host serves as a natural reservoir for *M*. *tuberculosis*, which is an intracellular obligate and aerobic bacillus that multiplies within macrophages. The bacterium triggers the production of free radicals and avoids being killed by the same radicals [1]. In the lungs, macrophages produce cytokines and chemokines that attract other phagocytic cells, including monocytes, other alveolar macrophages, and neutrophils, which eventually form a nodular granulomatous structure called a tubercle. The ability of *M*. *tuberculosis* to efficiently establish latent infection has enabled it to spread to nearly one-third of individuals worldwide.

Sarcoidosis is a systemic granulomatous disease characterized by the formation of epithelioid cell granulomas (and is accompanied by an infiltration of inflammatory cells) without caseous necrosis. Multisystem granulomatous inflammation involving the lungs, lymph nodes, skin, eyes, heart, and muscles is a hallmark of sarcoidosis. Infectious and genetic factors, as well as autoimmunity, are considered to be potential causes of SA [2–4]. In addition, mycobacterial antigens, such as the 6-kDa ESAT6, catalase—peroxidase (mKatG), superoxide dismutase A (Sod A) and *M*. *tuberculosis* heat shock proteins (Mtb-hsp) have been suggested to be infectious factors for the pathogenesis of SA [5].

In the middle of the 20th century, *M*. *tuberculosis* was isolated from several sarcoidosis patients by Scadding [6]. The relationship between *M*. *tuberculosis* and sarcoidosis has since been explored with increasing frequency. The advent of molecular biology invigorated the search for mycobacterial DNA in sarcoidosis patients [7, 8]. One meta-analysis conducted by Gupta [9] compiled 31 published studies on the presence of mycobacteria in sarcoidosis patients. The authors concluded that the evidence from pooled analysis favored the existence of an association between mycobacteria and sarcoidosis.

Recent studies evaluating immunological evidence of mycobacterial antigens in sarcoidosis patients has renewed interest in the role of mycobacteria in sarcoidosis [10], indicating that *M*. *tuberculosis* antigens could be involved in the pathogenesis of sarcoidosis. Many studies on the T-cell response to *M*. *tuberculosis* antigens have been conducted using an interferon gamma release assay (IGRA), which has been proven to be more efficient than the TST (tuberculin skin test) [11]. We conducted a meta-analysis of the available published literature to analyze the role of mycobacteria in the pathogenesis of sarcoidosis.

## Materials and Methods

### Search strategy and selection criteria

The aim of this meta-analysis was to collect all publicly available studies on the immune response to *M*. *tuberculosis* antigens in patients with sarcoidosis. To identify relevant articles for inclusion in this review, all authors independently searched PubMed, Embase, and Cochrane Library databases for relevant studies published from 1990–2015 using the following terms: sarcoidosis AND mycobacteria (OR) mycobacterium; sarcoidosis AND mycobacterium tuberculosis; and sarcoidosis AND tuberculosis. Additional eligible studies were identified by reviewing the references of all retrieved- literatures and review articles addressing the relationship between sarcoidosis and mycobacteria. The outcome measure of all included studies was positivity incidence of the immune response to *M*. *tuberculosis* antigens. No language or geographic restrictions were imposed on identified studies.

### Data abstraction and Quality Assessment

Paired reviewers (Chuling Fang and Hui Huang) independently evaluated studies for eligibility using a two-stage procedure. During the first stage, all identified abstracts were evaluated to ensure that they were involved in the relationship between sarcoidosis and mycobacteria. All potentially relevant studies were retrieved and selected for the second stage of the procedure, in which a full-text review was performed to determine whether the studies provided the positivity incidence of the immune response. Any disagreements were resolved by discussion and consensus. Data concerning the immune response to *M*. *tuberculosis* antigens were extracted onto standardized data collection forms by the two reviewers, and the verified data were entered into a Microsoft Excel spreadsheet (XP professional edition; Microsoft Corp, Redmond, WA, USA). The following items were extracted: 1) the publication details including the study geography (country or region), year of publication, first author and other citation details; 2) the number of patients with sarcoidosis and the type of mycobacterium (*M*. *tuberculosis* or other mycobacterium) used in analysis; 3) details of the technique used to evaluate the immune response in samples; 4) the health condition of the control subjects and whether details on TST outcomes were provided and 5) the numbers of the samples with an active immune response to mycobacterial antigens for patients with sarcoidosis and control. No attempt was made to include unpublished data. Methodological quality was assessed by the Newcastle-Ottawa Scale (NOS).

### Data Synthesis and Analysis

The included articles were analyzed using Cochrane Collaboration Review Manager statistical software (version 5.3; Cochrane Collaboration, Oxford, UK). To calculate the percentage of sarcoidosis samples with a positive immune reaction to *M*. *tuberculosis*, binomial proportions were used in which the numerator was sarcoid samples with active immune response to *M*. *tuberculosis* antigens and the denominator was the total number of study samples. As dichotomous outcomes, the percentage of sarcoid samples with a positive immune response out of the total samples versus the same percentage of control samples was expressed as an odds ratio (OR) and a 95% confidence interval (CI). The fixed-effects model was used, and the random-effects model weighted by the Mantel-Haenszel method was utilized for data with significant heterogeneity (P value of x^2^ test <0.05 and *I*^2^ >50%). P<0.05 was considered statistically significant.

### Assessment of heterogeneity

The impact of heterogeneity on the pooled estimates of individual outcomes of meta-analysis was assessed using the Chi-squared test and/or I^2^ test (this test measures the extent of inconsistency among studies’ results and is interpreted as the approximate proportion of total variation in study estimates due to heterogeneity rather than sampling error). An I^2^ value >50% indicates significant heterogeneity.

### Assessment of publication bias

A funnel plot test was used to assess publication bias. The funnel plot is a measurement of the log of the OR (on the X-axis; a measurement of diagnostic accuracy) against the standard error of the log of the OR (on the Y-axis; an indicator of sample size). Each point represents a study in meta-analysis.

## Results

### Study Identification

**Fig 1:**
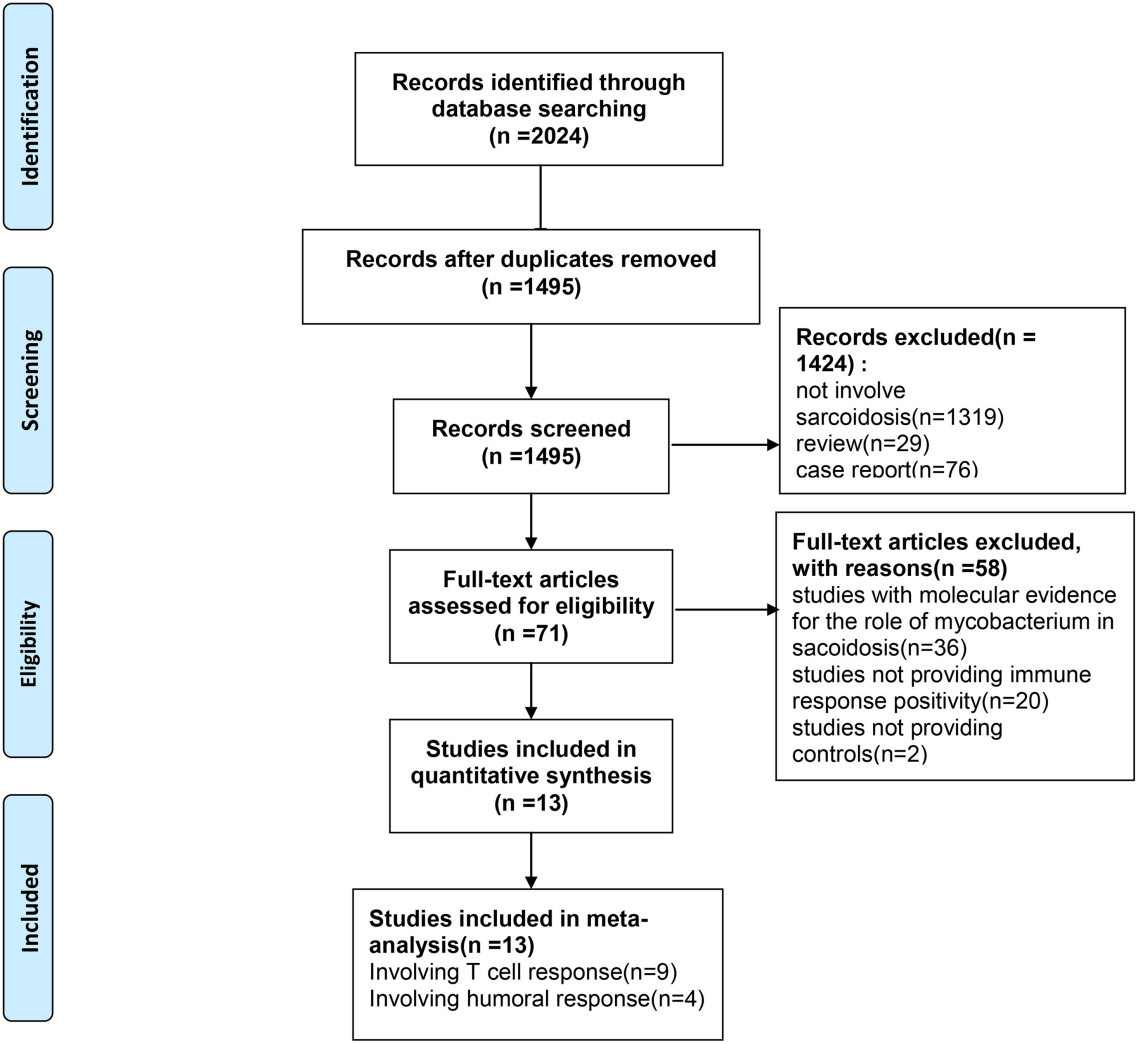
Flow diagram of assessment of studies identified in meta-analysis.

The process of identifying eligible studies is summarized in Fig 1. Studies were retrieved using the aforementioned process. After an evaluation of the title and abstract, 71 studies were subjected to further assessments. After full-text reviews, 13 trials (13articles) were included in meta-analysis, and related data were extracted. Detailed characteristics of the included studies are provided in Table 1. The 13 studies [12–24] published between 1996 and 2014 enrolled a total of 733 participants: 555 involving the T-cell response (253 in the sarcoidosis group, 302 in the control group with PPD-, unknown PPD status or PPD+ (LTBI)); and 178 Involving the humoral response (100 in the sarcoidosis group and 78 in the control group). Six of the trials were conducted in the USA [13, 16–19, 22], two were performed in India [14, 20], and one each was carried out in Germany [15], the Netherlands [12], Poland [21], Sweden [23] and Australia [24] respectively.

**Table 1:** Studies evaluating the role of mycobacteria in sarcoidosis using immunology analysis.

First author	Year	Site	Technique	Patients				Control		
				n	tissue/fluid	N	mycobacteria	n	diagnosis	N
**T-cell immune responses**										
Hofland	2014	Netherlands	IFN-γ ELISpot measuring responses to PPD	5	BALF	32	MTB	13	Other causes of interstitial lung disease, unknown PPD	86
Ahmadzai	2012	Australia	IFN-γ ELISpot measuring responses to ESAT-6 and KatG	10	Peripheral blood mononuclear cells (PBMCs)	16	MTB	4/5	Healthy, PPD-/ PPD+(LTBI)	17/5
Oswald-Richter	2012	USA	Flow cytometry measuring IFN-γ production in response to ESAT-6	17	BALF	27	MTB	2	Disease control, PPD-	14
Gupta	2011	India	QFT-GIT measuring responses to ESAT-6, CFP-10 and TB 7.7	13	PBMCs	38	MTB	5	Healthy, PPD<10mm	18
Horster	2009	Germany	IFN-γ ELISPOT measuring responses to ESAT-6	7	BALF	15	MTB	7	Bacterial and viral pneumonia, cryptogenic organizing pneumonia, and bronchogenic carcinoma, PPD≤15mm	29
Oswald-Richter	2009	USA	Flow cytometry measuring IFN-γproduction in responses to ESAT-6 and KatG	32	BALF	44	MTB	1	Disease control, PPD-	27
Drake	2007	USA	Flow cytometry measuring IFN-γproduction in responses to ESAT-6 and KatG	15	PBMCs	26	MTB	1/7	Healthy, PPD-/ PPD+ (LTBI)	24/8
Carlisle	2007	USA	ELISPOT assessing Th1 responses to sodA, mkatG and ESAT-6	12	PBMCs	30	MTB	1/6	Healthy, PPD-/ PPD+ (LTBI)	26/10
Hajizadeh	2007	USA	IFN-γ ELISPOT measuring responses to Antigen 85A	15	PBMCs	25	MTB	2/14	Healthy, PPD-/ PPD+ (LTBI)	22/16
**humoral immunity**										
Agarwal	2012	India	Humoral responses to ESAT-6 and CFP-10	11	blood samples	18	MTB	0	healthy, unknown PPD	20
Dubaniewicz	2006	Poland	Anti-Mtb-hsp70, -Mtb-hsp65 and -Mtb-hsp16 antibodies	12	blood samples	37	MTB	0	PPD-	18
Song	2005	USA	IgG antibodies to mKatG	12	PBMCs	25	MTB	0	healthy PPD−	11
El-Zaatari	1996	Sweden	Humoral reactivities to *M*. *paratuberculosis* recombinant clones expressing p36	7	blood samples	7	MPTB	13	Ulcerative colitis, noninflammatory bowel disease or healthy, unknown PPD	38

BALF = Bronchoalveolar lavage fluid, PBMCs = Peripheral blood mononuclear cells, LTBI = Latent tuberculosis infection.

All studies performed detailed immunology analysis and the meta-analysis included studies conducted around the world (Table 1). In total, the studies included 353 samples (235 blood samples and 118 BALF samples) from 353 patients with proven sarcoidosis as well as 380 samples (224 blood samples and 156 BALF samples) from 380 controls (with another lung disease or healthy with PPD-, PPD+ or unknown PPD status). Nine studies [12–19, 24] on T-cell immune response were included. Among them, the control group in two studies [13, 16] were composed of PPD- individuals, those in other three studies [12, 14, 15)] contained individuals with an unknown PPD status, and the remaining four [17–19, 24] used two control groups at the same time, consisted of PPD+ (LTBI) individuals and PPD- individuals respectively. Four studies [20–23] were included with humoral immune responses, the control groups of two studies [21, 22] among them were PPD-, and the other two studies [20, 23] were with unknown PPD subjects.

The quality scores of all 13 included studies were above five points, ranging from five to nine points. Five studies [12, 13, 16, 20, 24] achieved the highest score, and four others [14, 15, 18, 19] also achieved high score (Table 2). All studies were case control studies. All of the cases had adequate definitions with independent validation. Four studies [12, 13, 16, 20] controlled age and sex to insure the comparability of cases and controls. In addition, four studies [20–23] utilized three different methods (ELISA, immunohistochemical analysis, or protein immunoblotting) to test the humoral response to *M*.*tuberculosis* antigens. Further, nine studies [12–19, 24] used IGRA to test the T-cell response to these antigens.

**Table 2:** The Newcastle-Ottawa Scale (NOS) for assessing the quality of nonrandomized studies.

Non RCT studies		Selection			Comparability			Exposure			Total Quality score
First author	year	The case definition is adequate with independent validation	Consecutive or obviously representative series of cases	Community controls	Controls with no history of disease (endpoint)	Cases and controls with comparable ages	Cases and controls with comparability on any other factors	Ascertainment of exposure using secure records (eg surgical records) or structured interviews with blinding to case/control statuses	Ascertainment of exposure using the same method for cases and controls	Ascertainment of exposure with non-response rate for both groups	
**T-cell immune responses**											
**Hofland**	2014	*	*	*	*	*	*	*	*	*	9
**Ahmadzai**	2012	*	*	*	*	*	*	*	*	*	9
**Oswald-Richter**	2012	*	*	*	*	*	*	*	*	*	9
**Gupta**	2011	*	*	*	*			*	*	*	7
**Horster**	2009	*	*	*	*			*	*	*	7
**Oswald-Richter**	2009	*	*	*	*	*	*	*	*	*	9
**Drake**	2007	*	*	*	*			*	*	*	7
**Carlisle**	2007	*	*	*	*			*	*	*	7
**Hajizadeh**	2007	*			*			*	*	*	5
**Humoral immunity**											
**Agarwal**	2012	*	*	*	*	*	*	*	*	*	9
**Dubaniewicz**	2006	*			*			*	*	*	5
**Song**	2005	*			*			*	*	*	5
**El-Zaatari**	1996	*			*			*	*	*	5

one “*” means one point.

### T-cell immune response

**Fig 2:**
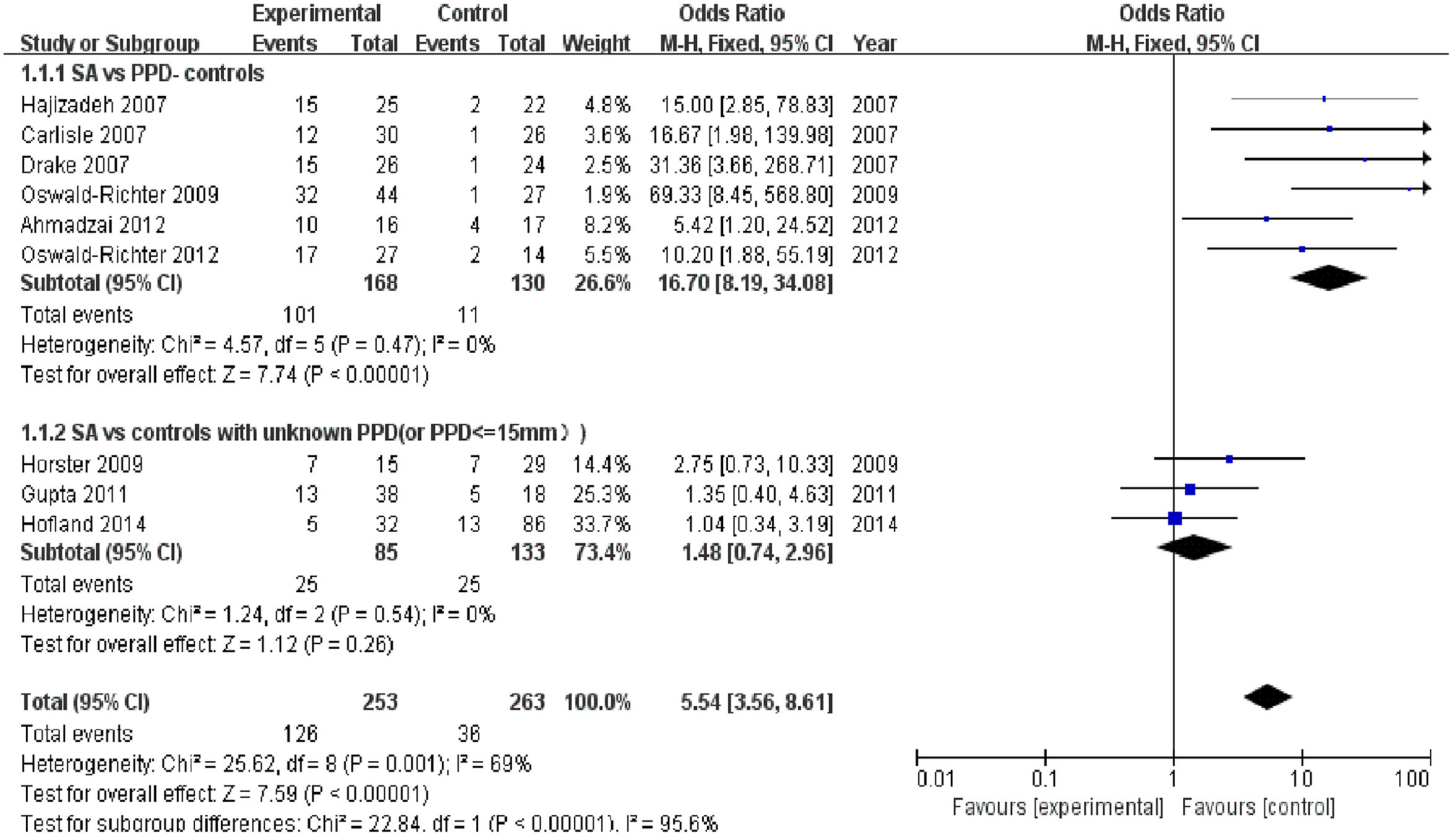
Forest plot of trials analyzing the positivity incidence of the T-cell response to *M*. *tuberculosis* antigens in sarcoidosis patients versus controls with PPD- and unknown PPD statuses.

**Fig 3:**
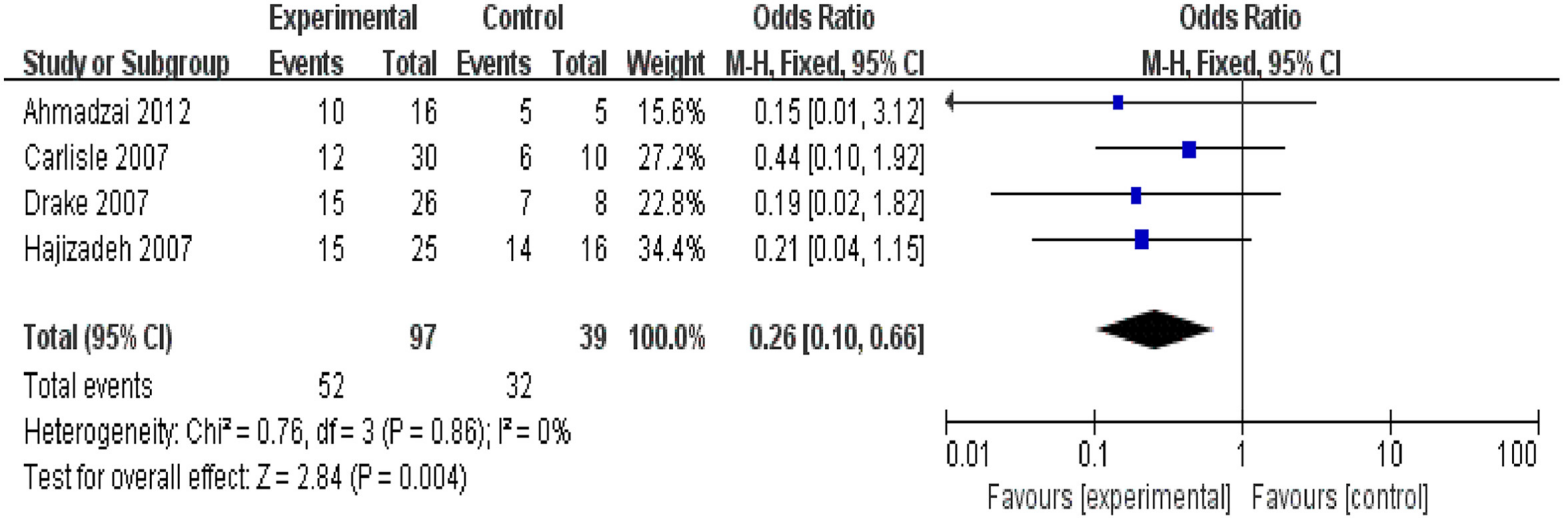
Forest plot of trials analyzing the positivity incidence of T-cell response to *M*. *tuberculosis* antigens in sarcoidosis patients versus PPD+ controls (LTBI). LTBI = latent tuberculosis infection.

**Fig 4:**
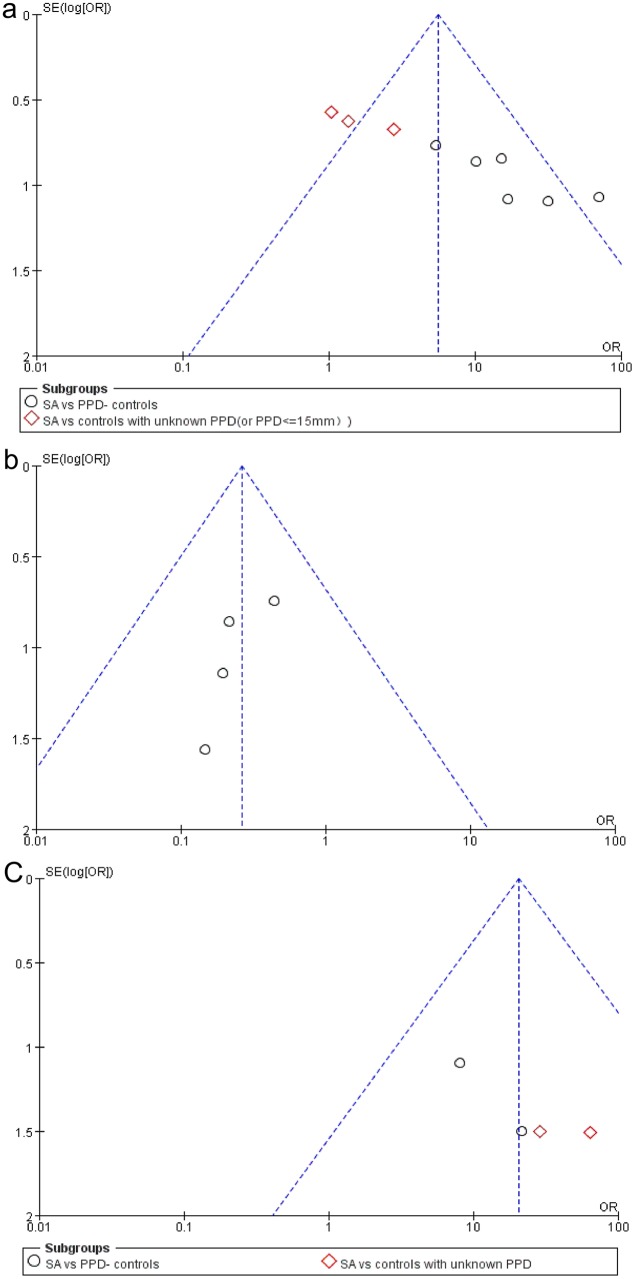
Funnel plot assessing publication bias. **a. Funnel plot for the studies on the T-cell response to *M*. *tuberculosis* antigens with controls of PPD- or unknown PPD status; b. Funnel plot for the studies on the T-cell response to *M*. *tuberculosis* antigens with PPD+ controls; c. Funnel plot for studies on the humoral response to *M*. *tuberculosis* antigens**. SA = sarcoidosis.

Nine trials [12–19, 24] reported the positivity incidence of the T-cell immune response to *M*. *tuberculosis* specific antigens. Among the 253 samples in the sarcoidosis group, 126 exhibited a positive immune response to *M*. *tuberculosis* specific antigens, as demonstrated by immunology. The combined results of the nine trials revealed that the sarcoidosis patients had a significantly higher positivity rate of the T-cell immune response to *M*. *tuberculosis* specific antigens compared to the controls with PPD- or unknown PPD status (OR = 5.54, 95% CI: 3.56–8.61, P<0.00001) (Fig 2). Combined analysis of six trials [13, 16–19, 24] revealed that the sarcoidosis patients had a significantly higher positivity rate than the subgroup of PPD- controls (OR = 16.70, 95% CI: 8.19–34.08, P<0.00001). Further, combined analysis of the three trials [12, 14, 15] revealed that the positivity rate of immune response in the sarcoidosis group did not significantly differ between the sarcoidosis patients and subgroup of controls with an unknown PPD status (OR = 1.48, 95% CI: 0.74–2.96, P = 0.26) (Fig 2). An additional combined analysis of four trials [17–19, 24] revealed that the sarcoidosis patients had significantly lower positivity rate than the PPD+ controls (LTBI) (OR = 0.26, 95% CI:0.10–0.66, P = 0.004) (Fig 3). Heterogeneity analysis revealed that there was no substantial heterogeneity in either the PPD- or unknown PPD control subgroups as assessed by using the I^2^ statistics (subgroup of PPD- controls (Q (d.f. = 5) = 4.57, P = 0.47, I^2^ = 0%); subgroup of controls with unknown PPD status (Q (d.f. = 2) = 1.24, P = 0.54, I^2^ = 0%)) (Fig 2). However, the heterogeneity between these two subgroups was substantial (Q (d.f. = 1) = 22.84, P<0.00001, I^2^ = 95.6%) (Fig 2). Further, no substantial heterogeneity was detected in the PPD+ control group (Q (d.f. = 3) = 0.76 P = 0.86, I^2^ = 0%) (Fig 3). Publication bias was not evident, as estimated by generating a funnel plot for the studies on the T-cell immune response to *M*. *tuberculosis* specific antigens (Fig 4a and 4b).

### Humoral response

**Fig 5:**
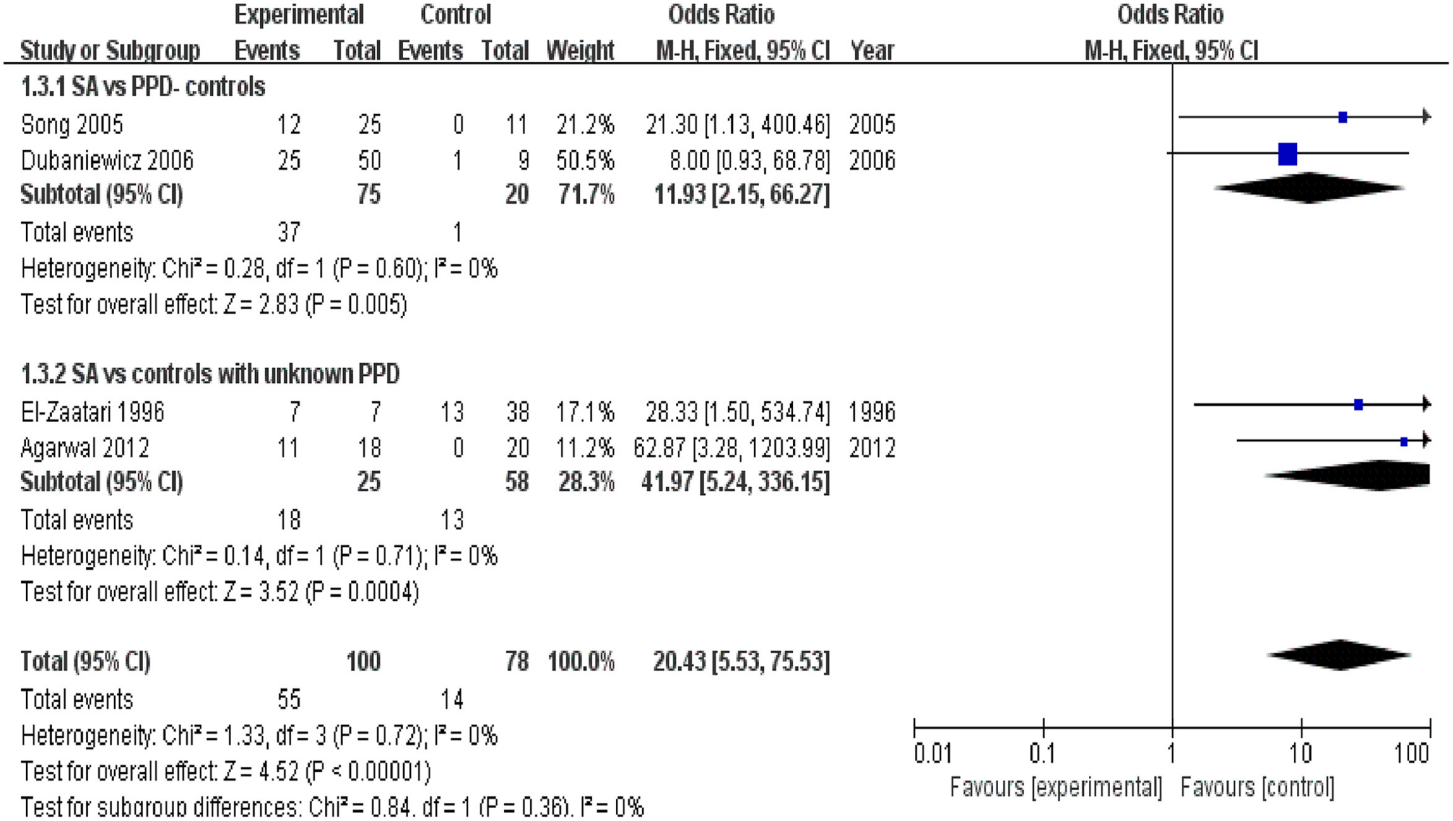
Forest plot of trials analyzing the positivity incidence of the humoral response to *M*. *tuberculosis* antigens in sarcoidosis patients versus controls.

Four trials [20–23] reported the positivity incidence of the humoral immune response to *M*. *tuberculosis* specific antigens. Of the100 samples in the sarcoidosis group, 55 exhibited a positive immune response to *M*. *tuberculosis* specific antigens, as demonstrated by ELISA and immunohistochemical analysis. The combined results of four trials [20–23] revealed that the sarcoidosis group had a significantly higher positivity rate of humoral response to *M*. *tuberculosis* specific antigens than the control group (OR = 20.43, 95% CI: 5.53–75.53, P<0.0001). Further, the combined results of two trials [21, 22] revealed that the sarcoidosis group had a significantly higher positivity rate of the humoral immune response to *M*. *tuberculosis* specific antigens than subgroup of PPD- controls (OR = 11.93, 95% CI: 2.15–66.27, P = 0.005). Similarly, combined analysis of another two trials [20, 23] revealed that the immune response positivity rate in the sarcoidosis group was significantly higher than that in the subgroup of controls with an unknown PPD status (OR = 41.97, 95% CI: 5.24–336.15, P = 0.0004) (Fig 5). Heterogeneity test revealed that there was not substantial heterogeneity in either of these two subgroups, as assessed using the I^2^ statistic (subgroup of PPD- controls (Q (d.f. = 1) = 0.28, P = 0.60, I^2^ = 0%); subgroup of controls with unknown PPD status (Q (d.f. = 1) = 0.14, P = 0.71, I^2^ = 0%)) (Fig 5). The heterogeneity between these two subgroups was also not substantial (Q (d.f. = 1) = 0.84, P = 0.36, I^2^ = 0%). Publication bias was not evident, as estimated by generation of a funnel plot for the studies on the T-cell immune response to *M*. *tuberculosis* specific antigens (Fig 4c).

## Discussion

Meta-analysis was the main method used in this research paper. It is more accurate and reliable than regression analysis or original papers.

The relationship between sarcoidosis and *M*. *tuberculosis* has been a highly contested and challenging issue. Further analysis is important considering their opposite therapeutic effects In the current meta-analysis, a greater positivity incidence of the immune response to *M*. *tuberculosis* specific antigens was observed in sarcoidosis group compared to the control group of subjects with PPD- or unknown PPD status for both the T-cell immune response and humoral response, indicating that these antigens induce T-cell-specific responses in the blood or BALF of sarcoidosis patients. However, it is important to note that sarcoidosis patients may be more likely to have positive TST results (i.e. to be more sensitive to LTBI) than healthy individuals due to unknown host factors with no known etiologic link between sarcoidosis and *M*. *tuberculosis*. Notably, the positivity incidence of T-cell immune response to *M*. *tuberculosis* specific antigens in sarcoidosis group was found to be significantly lower in the sarcoidosis patients than in the PPD+ (LTBI) controls, although the incidences did not differ among the four primary trials. Further, it was hypothesized that *M*. *tuberculosis* specific antigens response would not be observed in all sarcoidosis patients, and that the specific mycobacterial antigen may represent just one of the most important factors for the pathogenesis of sarcoidosis.

In the T-cell immune response, no significant heterogeneity was observed in either the subgroup of PPD- controls or in the subgroup of controls with an unknown PPD status. Similarly, no significant heterogeneity was detected in the group of PPD+ (LTBI) controls. Similarly, no significant heterogeneity was detected in the group of PPD+ (LTBI) controls. Further, in the humoral response, no significant heterogeneity was observed in either the subgroup of PPD- controls or the subgroup of controls with an unknown PPD status. However, significant heterogeneity was detected between the two subgroups with PPD- or unknown PPD status on the T-cell immune response, indicating that PPD status strongly influenced experimental outcome.

A number of original studies have examined mycobacteria in samples of sarcoidosis patients [7, 25–32], with different results. In addition, a meta-analysis [9] on the molecular evidence of the role of mycobacteria in sarcoidosis conducted in 2007 demonstrated mycobacterial presence in sarcoidosis lesions, suggesting the presence of an association between mycobacteria and sarcoidosis in some cases. However, it is important to emphasize that the mere presence of mycobacterial DNA does not indicate the existence of a cause-and-effect relationship. In 1998, evidence that the granulomatous response is driven by tissue antigens in sarcoidosis was provided by analysis of TCR expression [33]. Many recent studies have reported that *M*. *tuberculosis* antigens are involved in the pathogenesis of sarcoidosis [10, 34]. Among our included studies, one study conducted by Song reported the detection of several *M*. *tuberculosis* antigens including catalase‑peroxidase antigen (mKatG) and Mtb 16S rRNA in nearly 40% of their sarcoidosis subjects as detected by *in situ hybridization* [22]. Further, some studies have suggested that some antigens of *M*. *tuberculosis* (and not the whole mycobacteria) are involved in the pathogenesis of sarcoidosis [5, 16]. These data suggest the presence of mycobacterial proteins preferentially in sarcoidosis patients. Indeed, some factors do favor the involvement of mycobacteria in sarcoidosis, including the following: (1) histopathological appearances of the granulomas; (2) an immune response to *M*. *tuberculosis* antigens in sarcoidosis patients; (3) development of mycobacterial disease either coincidentally, before or after development of sarcoidosis [35–37]; (4) the detection of mycobacteria from samples of patients with sarcoidosis by PCR [26, 27]; and (5) acid-fast cell wall-deficient forms (CWDF) of bacteria can be grown from the blood of patients with sarcoidosis [38, 39]. It is important to note that mycobacterial (TB or NTM) antigens and not the active mycobacteria may play roles in the pathogenesis of sarcoidosis. One hypothesis is that an impediment in the removal of poorly degraded antigenic material may contribute to the pathogenesis of sarcoidosis [22, 40]. However, it is important to note that mycobacterial antigens alone cannot explain the pathogenesis of this disease in all subjects, as no immune response to *M*. *tuberculosis* antigens or molecular evidence was observed in some sarcoidosis subjects. Therefore, we assumed that other factors besides mycobacterial antigens contribute to the pathogenesis of sarcoidosis.

There are several limitations to this study. First, although data were collected from more than 700 patients, the number of incorporated studies was still small, and therefore these results may have been affected by publication bias. Second, the definite sensitivity of the immunological data is unknown, as they were not described or analyzed in detail in this study. Third, some variations may also have occurred due to differences in local environmental exposure and the fact that some sarcoidosis patients may have a disorder initiated by organisms or triggers other than mycobacteria. Therefore, additional studies would be useful to provide more concrete data to investigate the accuracy of this conclusion are needed.

In conclusion, the results of this meta-analysis have demonstrated an association between mycobacteria (especially *M*.*tuberculosis*) and sarcoidosis. Mycobacteria was not observed in all cases of sarcoidosis, however, these bacteria indeed represent an important factor involved in the pathogenesis of this disease. It has been hypothesized that only some specific, poorly degraded mycobacterial pathogenic antigens contribute to immune-mediated granulomatous inflammation rather than the whole active mycobacteria and that they elicit a type IV immune response.

## Supporting Information

S1 Table PRISMA checklist of this meta-analysis. (DOC)
